# Metabolic Regulation of Ferroptosis in Cancer

**DOI:** 10.3390/biology10020083

**Published:** 2021-01-22

**Authors:** Min Ji Kim, Greg Jiho Yun, Sung Eun Kim

**Affiliations:** 1 Department of Biosystems and Biomedical Sciences, College of Health Sciences, Korea University, Seoul 305-350, Korea; mkmj06@korea.ac.kr (M.J.K.); blueshark19@korea.ac.kr (G.J.Y.); 2 Department of Integrated Biomedical and Life Sciences, College of Health Sciences, Korea University, Seoul 305-350, Korea

**Keywords:** ferroptosis, iron metabolism, cyst(e)ine metabolism, SLC7A11, glutathione metabolism, GPX4, lipid peroxidation, reactive oxygen species

## Abstract

**Simple Summary:**

Ferroptosis is a recently defined nonapoptotic form of cell death that is associated with various human diseases, including cancer. As ferroptosis is caused by an overdose of lipid peroxidation resulting from dysregulation of the cellular antioxidant system, it is inherently closely associated with cellular metabolism. Here, we provide an updated review of the recent studies that have shown mechanisms of metabolic regulation of ferroptosis in the context of cancer.

**Abstract:**

Ferroptosis is a unique cell death mechanism that is executed by the excessive accumulation of lipid peroxidation in cells. The relevance of ferroptosis in multiple human diseases such as neurodegeneration, organ damage, and cancer is becoming increasingly evident. As ferroptosis is deeply intertwined with metabolic pathways such as iron, cyst(e)ine, glutathione, and lipid metabolism, a better understanding of how ferroptosis is regulated by these pathways will enable the precise utilization or prevention of ferroptosis for therapeutic uses. In this review, we present an update of the mechanisms underlying diverse metabolic pathways that can regulate ferroptosis in cancer.

## 1. Introduction

“Every true story ends in death” are the words of Ernest Hemingway. Cell death is an important aspect of mammalian development and homeostasis and is tightly integrated with the physiological and pathological state of an organism. The proper orchestration of cell death both spatially and temporally is critical for development and, consequently, the malfunction of cell death mechanisms can contribute to various human diseases. Ferroptosis is a nonapoptotic cell death mechanism characterized by iron-dependent accumulation of lipid peroxides. Importantly, ferroptosis has been implicated in neurodegenerative diseases, kidney degeneration, and ischemic injury in multiple organs. Studies using ferroptosis inhibitors have shown to be effective in ischemia/reperfusion-induced damage and in models of Huntington’s disease and Parkinson’s disease, suggesting that ferroptosis is a promising therapeutic target for the treatment of these diseases. On the other hand, the possibility of harnessing ferroptosis as a method for cancer therapy is also gaining much attraction. Indeed, the original study that led to the identification of erastin, a ferroptosis-inducing drug, was aimed for the selective killing of RAS-mutant cancer cells [1]. Since then, multiple lines of research have suggested the important role of ferroptosis in cancer development and treatment. For example, ferroptosis has been suggested to be a critical factor for the activities of the tumor suppressors p53, BRCA1-associated protein 1 (BAP1), and fumarase [2,3,4]. Ferroptosis can also play a tumor-suppressive role during the metastasis of cancer cells as melanoma cells in the lymph are protected from ferroptosis and, thus, can form more metastases than those in the blood [5]. Similarly, circulating tumor cells from melanoma patients have been shown to regulate lipogenesis and iron homeostasis pathways to confer resistance to ferroptosis [6]. 

Several types of cancer cells are susceptible to ferroptosis; thus, ferroptosis may represent a novel mode of anticancer therapy. Cancer types that show high sensitivity to ferroptosis include renal cell carcinoma, diffuse large B cell lymphoma, adrenocortical carcinoma, and ovarian cancer [7,8,9]. Recently, it was reported that therapy-resistant or drug-tolerant cells, which are represented by a high mesenchymal state, depend on the glutathione peroxidase 4 (GPX4) pathway to evade ferroptosis, suggesting that ferroptosis induction represents an anticancer strategy for these therapy resistant cancer cells [10,11]. Similarly, platinum-tolerant or cisplatin-resistant cancer cells were also shown to exhibit increased vulnerability to ferroptosis [12,13]. This was also shown in the case of melanoma, where the dedifferentiation state of these cells was shown to correlate with increased sensitivity to ferroptosis [14]. The function of ferroptosis in cancer can also involve multiple cell types in the tumor microenvironment. A recent study shows that neutrophils can induce ferroptosis in glioblastoma cells by transferring myeloperoxidase-containing granules into the cancer cells [15]. Additionally, immunotherapy activated CD8^+^ T cells can induce ferroptosis in cancer cells by interferon gamma-mediated downregulation of the two subunits of system X_c_^−^, solute carrier family 7 member 11 (SLC7A11) and SLC3A2, leading to a decrease in the antioxidant capacity of cancer cells [16]. This effect can also synergize with radiotherapy through independent SLC7A11 suppression by the ataxia-telangiectasia mutated (ATM) serine/threonine kinase [17].

For the induction of ferroptosis in cancer, agents that inhibit cystine uptake via the cystine/glutamate antiporter, system X_c_^−^, such as sulfasalazine, can induce ferroptosis and arrest tumor growth [18]. The multikinase inhibitor sorafenib, which is used for advanced hepatocellular carcinoma, has also been shown to induce ferroptosis by inhibiting system X_c_^−^ [19]. Similarly, the systemic depletion of cyst(e)ine using an engineered cyst(e)ine-degrading enzyme conjugate can trigger ferroptosis and arrest tumor growth [20] and the antimalarial drug, artesunate, has also been identified as an activator of ferroptosis [21]. Furthermore, ferroptosis can synergize with cisplatin to increase cytotoxicity in cancer cells [22] and radiation therapy can also synergize with ferroptosis inducers [23,24]. Although several ferroptosis-inducing agents exist, including erastin, RSL3, and ML210, further studies to enhance the pharmacokinetic properties are needed for use in clinical settings [7,25].

In essence, ferroptosis is a process that occurs through metabolic dysregulation. Ferroptosis is modulated by perturbation of lipid repair systems involving glutathione and GPX4, the enzyme that converts toxic lipid hydroperoxides to nontoxic lipid alcohols. The term ferroptosis was coined in 2012, describing the cell death induced by erastin, that inhibits the import of cystine and leads to the depletion of glutathione and inactivation of GPX4 [26]. Inactivation of GPX4 through the depletion of glutathione or through direct GPX4 inhibition results in the accumulation of lipid peroxidation that leads to cell death. Ferroptosis can be suppressed by iron chelators, lipophilic antioxidants, lipid peroxidation inhibitors, or the depletion of polyunsaturated fatty acids (PUFAs). Thus, ferroptosis is characterized by the perturbation of an intricate metabolic network which we will describe in detail in the following sections.

## 2. Iron Metabolism

**Figure 1:**
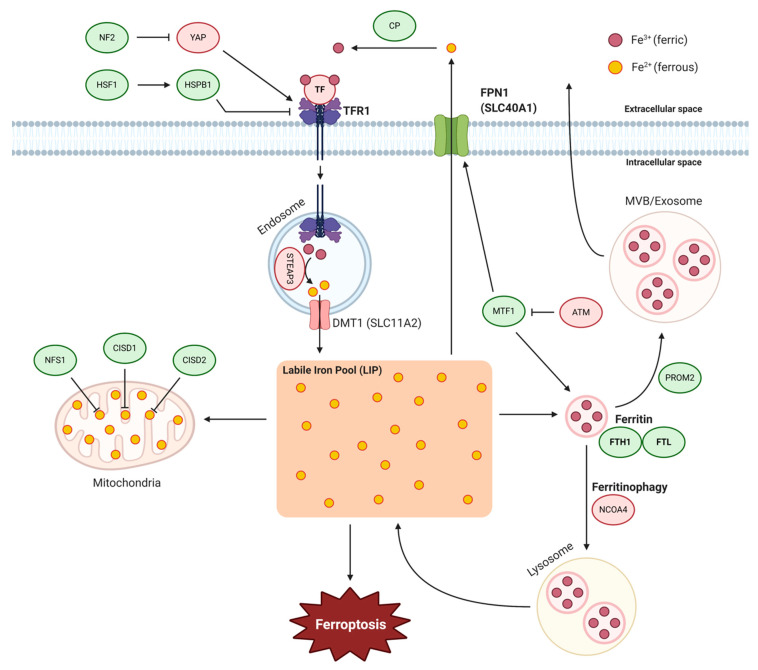
Iron metabolism regulates ferroptosis. Ferric iron (Fe^3+^, red circle) bound to TF can form a complex with TFR1 to be endocytosed into cells. In the endosome, ferric iron is reduced to ferrous iron (Fe^2+^, yellow circle) by STEAP3 and transported into the cytoplasm by DMT1 or SLC11A2. Ferrous iron is stored in ferritin heteropolymers to protect cells and tissues from iron-mediated damage. Ferritin can undergo ferritinophagy, an autophagic degradation process, or exported out of the cell as MVB/exosomes. In the mitochondria, iron-sulfur clusters can be used by iron-sulfur proteins in various processes. Dysregulation in any of the above processes can lead to an increase in the labile iron pool and contribute to ferroptosis. Ferroptosis inhibiting and inducing factors are indicated in green and red, respectively. (Abbreviations: ATM, ATM serine/threonine kinase; CISD1/2, CDGSH iron-sulfur domain-containing protein 1/2; CP, ceruloplasmin; DMT1, divalent metal transporter 1; FPN1, ferroportin; FTH1, ferritin heavy chain 1; FTL, ferritin light chain; HSF1, heat shock factor 1; HSPB1, heat shock protein beta-1; MTF1, metal-regulatory transcription factor 1; MVB, multivesicular bodies; NCOA4, nuclear receptor coactivator 4; NF2, neurofibromin 2; NFS1, NFS1 cysteine desulfurase; STEAP3, six transmembrane epithelial antigen of the prostate 3; TF, transferrin; TFR1, transferrin receptor 1; YAP, yes-associated protein).

As the name ‘ferroptosis’ infers, the requirement of intracellular iron is a fundamental property of ferroptosis. As an important trace element in the human body, iron regulates numerous biological processes and, therefore, dysregulation of iron content or distribution can result in intracellular iron accumulation, which can lead to the damage of cells, tissues, and organs. Elaborate regulatory systems have evolved to maintain iron levels at sufficient yet safe concentrations in cells. Such homeostasis of iron metabolism is achieved by the coordination of iron uptake, utilization, recycling, storage, and export [27]. The iron dependency of ferroptosis was initially demonstrated by the use of iron chelators to inhibit ferroptosis. Additionally, in the same study, iron responsive element binding protein 2 (IRP2), a master regulator of iron metabolism, was found in an erastin-resistance screen establishing that ferroptosis is intricately modulated by iron metabolism. Perturbation of iron metabolism can be triggered in multiple ways such as direct oxidation of iron by FINO_2_, a peroxide-containing compound, or iron uptake with ultrasmall nanoparticles [28,29]. Similarly, salinomycin, an antibacterial drug, can also trigger ferroptosis by sequestering of iron in lysosomes [30]. Although the exact mechanisms through which cellular iron functions to facilitate ferroptosis remain unclear, cellular iron homeostasis is recognized as a key regulating factor in ferroptosis. Thus, any change in the import/export, storage, or turnover of iron has the potential of impacting ferroptosis sensitivity. Here, we discuss the processes of iron metabolism relevant to ferroptosis and how multiple mechanisms can influence ferroptosis in cancer (Figure 1).

### 2.1. Iron Import

Extracellular iron binds to transferrin (TF) to be taken up into cells through the transferrin receptor 1 (TFR1). TFR1 is abundantly expressed and involved in the progression of several cancer types, including brain, breast, colon, and liver cancers. The increased need for iron uptake leads to the high expression of TFR1, because iron is required for tumor cell proliferation in some cases [31]. However, TF and TFR1 are a positive regulator of ferroptosis, and in RAS-mutant cells, increased TFR1 expression contributes to ferroptosis sensitivity by increasing iron uptake whereas cells with knockdown of TFR1 become more resistant to erastin-induced ferroptosis [1,26]. This suggests that increased iron import from the extracellular environment can contribute to ferroptosis by augmenting the labile redox-active iron pool, which is important for ferroptosis. TFR1, along with acyl-CoA synthetase long-chain family member 4 (ACSL4), was shown to be a target of the YAP-TEAD complex, which can be inhibited by the tumor suppressor neurofibromin 2 (NF2), and similarly regulated non-cell-autonomously by cell density-dependent mechanisms [32]. Additionally, heat shock protein beta-1 (HSPB1) can reduce intracellular iron concentrations by inhibiting TFR1 expression [33]. Inhibition of the heat shock factor 1 (HSF1)-HSPB1 pathway or HSPB1 phosphorylation increases the anticancer activity of erastin in xenograft mouse models. TFR1 has been suggested as a ferroptosis-selective marker as TFR1-recognizing antibodies were shown to have selectivity in detecting cells undergoing ferroptosis [34]. In addition to the potential use of TFR1 as a ferroptosis marker, TFR1 was shown to accumulate on the cell surface during ferroptosis. This was most likely not through a disruption of clathrin-mediated endocytosis as the epidermal growth factor receptor was still internalized during ferroptosis. How TFR1 accumulates on the plasma membrane and whether this has a specific function in ferroptosis are questions for future studies. Interestingly, in contrast to the previous findings of TF having a positive role in ferroptosis, a recent study showed that hepatocyte-specific TF knockout mice have higher susceptibility to ferroptosis in liver fibrosis [35]. Patients with liver cirrhosis were shown to have reduced hepatic TF and increased hepatic iron levels suggesting that TF has a protective role in these contexts. It remains to be seen how TF or TFR1 function mechanistically in this scenario.

### 2.2. Ferritin and Ferritinophagy

Iron can exist in two forms, ferrous (Fe^2+^) or ferric (Fe^3+^), and most of the ferric form is incorporated into proteins or stored by ferritin so that there is a minimal amount of free iron in the labile iron pool (LIP). Ferritin is composed of ferritin light chain (FTL) and ferritin heavy chain 1 (FTH1). In RAS-mutant cells, increased TFR1 and decreased ferritin expression contribute to ferroptosis sensitivity by increasing iron uptake and decreasing iron storage, respectively [1]. Conversely, inhibiting the expression of IRP2, the major regulator of iron metabolism, can significantly increase the expression of FTL and FTH1, thereby inhibiting ferroptosis induced by erastin [26]. In a recent study, the DNA damage response serine/threonine kinase ATM was found to induce ferroptosis by inhibiting the expression of FTH1 and FTL, along with ferroportin (FPN1), which exports iron out of cells [36]. The coordinated changes of these iron regulators during ATM inhibition resulted in a lowering of the LIP and prevented erastin-induced ferroptosis. Furthermore, ATM inhibition enhanced the nuclear translocation of metal-regulatory transcription factor 1 (MTF1) and genetic depletion of MTF1 abolished the regulation of iron-regulatory elements by ATM and resensitized cells to ferroptosis. The mitochondrial ferritin (FtMt), which is structurally similar to cytosolic FTH1, also has a protective role in erastin-induced ferroptosis [37]. Overexpression of FtMt blocked the increase of LIP and ROS caused by erastin and led to prolonged survival of a ferroptosis model in *Drosophila*.

The cytosolic ferritin levels in cells can be regulated by a selective autophagy process called ferritinophagy, which involves the degradation of ferritin and consequent release of free iron [38]. At the genetic level, multiple autophagy-related genes have been identified as positive regulators of ferroptosis [39,40] and knockdown of nuclear receptor coactivator 4 (NCOA4), the specific cargo receptor, also inhibits ferritinophagy and ferroptosis [38]. These results suggest that autophagy activation can lead to ferritinophagy and promote ferroptosis by regulating iron homeostasis in cells. Additionally, an autophagy-independent mechanism of ferritin degradation was shown in a recent study. Dihydroartemisinin (DAT), a derivative of the antimalarial drug artemisinin, was found to sensitize cancer cells to ferroptosis by inducing lysosomal degradation of ferritin in an autophagy-independent manner [41]. Through these mechanisms, DAT can augment GPX4 inhibition-induced ferroptosis in cancer cells that were otherwise highly resistant to ferroptosis.

### 2.3. Iron–Sulfur Clusters

Iron can be used in cells upon assembly into iron–sulfur clusters which are required for the function of iron–sulfur proteins in a wide range of activities, including the electron transport chain, photosynthesis, or DNA repair. In a recent study, it was found that lung adenocarcinoma cells depend on high levels of the iron–sulfur cluster biosynthesis enzyme cysteine desulfurase (NFS1) to limit reactive iron that can cause lipid peroxidation [42]. NFS1 activity was particularly important for the survival in a high oxygen environment and resistance to ferroptosis in response to oxidative damage. In two other studies, members of the CDGSH iron–sulfur domain (CISD) protein family, CISD1 and CISD2, were also shown to negatively regulate ferroptosis by limiting mitochondrial iron uptake [43,44]. However, treatment of pioglitazone, which stabilizes CISD1 and CISD2, had opposing effects in these two studies, suggesting there are additional mechanisms to how CISD1 and CISD2 function to regulate ferroptosis.

### 2.4. Iron Export

When there is excess intracellular labile iron in the cell, the membrane protein FPN1 can export iron to maintain intracellular and plasma iron homeostasis. Hepcidin, a small peptide hormone secreted by hepatocytes, can promote the degradation of FPN1 and this has been shown to regulate erastin-induced ferroptosis in neuroblastoma cells [45]. As mentioned above, FPN1 regulation by ATM, through MTF1, can regulate ferroptosis [31]. Dysfunction of the iron export mechanism can regulate ferroptosis as shown by the example of siramesine, a lysosome disrupting agent, and lapatinib, a tyrosine kinase inhibitor, which can synergistically induce ferroptosis by decreasing FPN1 expression [46]. Another recently discovered mechanism of iron export is through prominin 2 (PROM2), a pentaspanin protein involved in lipid dynamics, which facilitates ferroptosis resistance by promoting the formation of ferritin-containing multivesicular bodies and exosomes that transport iron out of the cell [47]. These findings reveal that ferroptosis resistance can be driven by multiple iron export mechanisms. The function of ceruloplasmin (CP), a ferroxidase enzyme, is important for the oxidization of extracellular ferrous iron to ferric iron, which allows complex formation with TF and endocytosis into cells through TFR1 binding. The depletion of CP leads to the accumulation of intracellular ferrous iron and lipid reactive oxygen species (ROS), which promotes erastin- and RSL3-induced ferroptosis [48]. However, the effects of CP are dependent on FPN1 and were overridden by the effects of TFR1 and FPN1, suggesting that CP has minimal effects compared to the iron import and export regulated by TFR1 and FPN1, respectively.

## 3. Cyst(e)ine-Glutathione-GPX4 Axis

**Figure 2:**
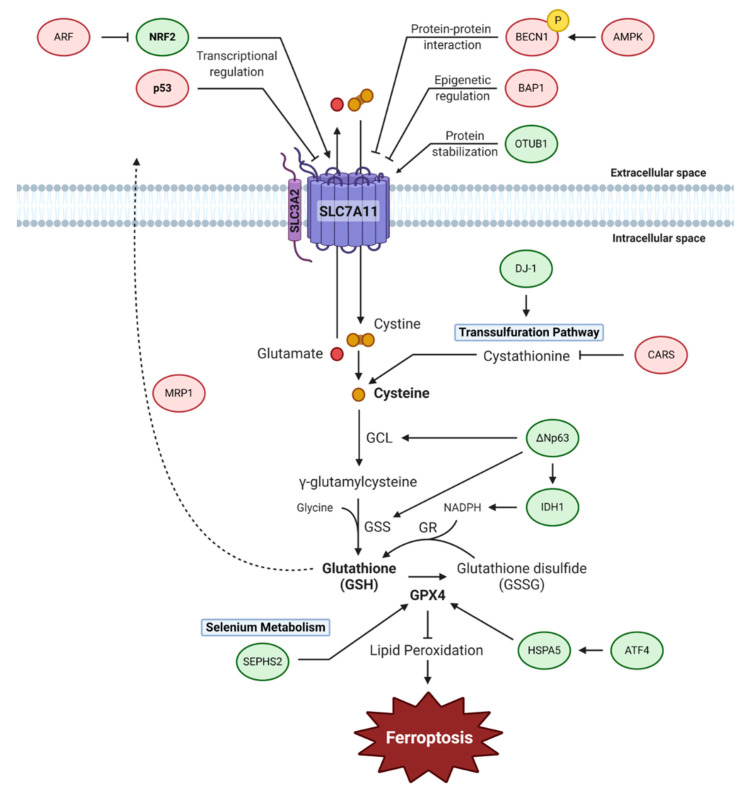
The cyst(e)ine-glutathione-GPX4 axis regulates ferroptosis. The cystine/glutamate antiporter, system X_c_^−^, is composed of two subunits, SLC7A11 and SLC3A2. System X_c_^−^ is the dominant pathway for regulating cysteine levels within cells by transporting cystine into cells. An alternate pathway for synthesizing cysteine is through the transsulfuration pathway. Cysteine is used as a substrate for glutathione biosynthesis, which is further used to activate GPX4 to inhibit lipid peroxidation. This antioxidative activity of GPX4 allows for the prevention of ferroptosis. Selenium metabolism is important for the activity of GPX4, which is a selenoprotein. Ferroptosis inhibiting and inducing factors are indicated in green and red, respectively. (Abbreviations: AMPK, AMP-activated protein kinase; ARF, alternative reading frame; ATF4, activating transcription factor 4; BAP1, BRCA1-associated protein 1; BECN1, beclin 1; CARS, cysteinyl-tRNA synthetase; DJ-1/PARK7, cancer- and Parkinson’s disease (PD)-associated protein; GCL, glutamate-cysteine ligase; GPX4, glutathione peroxidase 4; GR, glutathione reductase; GSS, glutathione synthetase; HSPA5, heat shock protein family A member 5; IDH1, isocitrate dehydrogenase 1; NADPH, nicotinamide adenine dinucleotide phosphate; ΔNp63, N-terminal truncated isoform of p63; NRF2, nuclear factor erythroid 2–related factor 2; OTUB1, OTU deubiquitinase, ubiquitin aldehyde binding 1; SEPHS2, selenophosphate synthetase 2; SLC3A2, solute carrier family 3 member 2; SLC7A11, solute carrier family 7 member 11).

The cyst(e)ine-glutathione-GPX4 axis functions as the main cellular pathway that protects cells from undergoing ferroptosis. Cystine, the oxidized form of cysteine, is predominantly present in the extracellular space, compared to cysteine which is prevalently intracellular due to the reducing conditions within cells [49]. These amino acids are essential for regulating the redox conditions both inside and outside of the cell and also act as a substrate for glutathione biosynthesis. Intracellular cysteine levels are mainly regulated by system X_c_^−^, also known as system xCT, which is a cystine/glutamate antiporter that transports a single cystine molecule into the cell in exchange for one molecule of glutamate. Due to its high utility, cysteine levels may also be regulated by the transsulfuration pathway, a system X_c_^−^-independent mechanism, by using methionine as a precursor [50]. Cysteine is used for glutathione biosynthesis, in a two-step cascade by the rate-limiting enzymes, γ-glutamylcysteine ligase (GCL) and glutathione synthetase (GSS) [51]. GCL is responsible for initiating the specific γ-ligation between glutamate and cysteine to form a dipeptide which is then used by GSS to form a peptide bond with glycine to generate glutathione. GPX4 is a glutathione-utilizing enzyme that prevents the accumulation of toxic lipid hydroperoxides by catalyzing their reduction into corresponding alcohols. Among the GPX family members, GPX4 is the only enzyme capable of this activity, making it a central regulator of ferroptosis. In the following section, we discuss how cysteine metabolism, glutathione metabolism, and GPX4 activity regulate ferroptosis (Figure 2).

### 3.1. System X_c_^−^

System X_c_^−^ is composed of two subunits; SLC3A2 and SLC7A11 [52]. SLC7A11 is in charge of the primary transport activity of the cystine/glutamate antiporter, while SLC3A2 maintains the protein stability of SLC7A11. When, and if, the function of SLC7A11 is inhibited or blocked, system X_c_^−^ would not be able to function normally and cause a breakdown in the balance of cysteine/cystine and glutamate concentrations. Therefore, SLC7A11 is one of the main negative regulators of ferroptosis and this has been recently confirmed by in vivo evidence of SLC7A11 depletion leading to ferroptosis in pancreatic cancer [53]. Several studies have also established that system X_c_^−^ may be capable of regulating the redox cycle independently of glutathione by working through the alanine/serine/cysteine transport cycle [49,54]. The three steps of the cycle are the transportation of cystine into cells, reduction of cystine into cysteine by cystine reductase, and the release of excess cysteine. The released cysteine can be oxidized into cystine in the extracellular space to be transported back into the cell.

Although it has been thought that the tumor suppressor function of p53 is mediated by apoptosis, senescence, and growth arrest, several mouse models suggested that tumor suppression can be achieved in the absence of these canonical functions but through the metabolic activities of p53. For example, whereas p53^3KR^, an acetylation-defective mutant at three acetylation sites, K116, K161, and K162, cannot elicit apoptosis, senescence, or growth arrest, it retains tumor suppressive functions [55]. It was shown that *SLC7A11* is a target gene of p53 and tumor suppression induced by p53^3KR^ was largely abrogated with SLC7A11 overexpression, demonstrating that ferroptosis plays an important role in the tumor suppressive activities of p53 [2]. Furthermore, an additional mutation at the acetylation site lysine K98 that leads to the complete abrogation of metabolic targets of p53, including SLC7A11 and TIGAR, is severely defective in suppressing tumor growth in mouse xenograft models [56]. In contrast, in the presence of wild-type p53, stabilization of p53 leads to the delayed induction of ferroptosis in cancer cells [57]. This happens even in the presence of p53-mediated SLC7A11 inhibition which would normally accelerate ferroptosis, suggesting that other mechanisms counterbalance this process. Further results showed that this delay in ferroptosis involves both a decrease in the rate of glutathione depletion and reduced accumulation of lipid ROS which requires the activity of the p53 transcriptional target, cyclin-dependent kinase inhibitor 1A (CDKN1A). Although these results seem to contradict each other, the acetylation-defective p53 mutants are unable to induce CDKN1A and, therefore, it is possible that both SLC7A11 inhibition and CDKN1A induction may happen concurrently in the presence of wild-type p53 and how this affects ferroptosis in various contexts will be of interest for future studies.

Another well-known regulator of SLC7A11 expression is nuclear factor E2-related factor 2 (NRF2), the main mediator of redox homeostasis. The treatment of ferroptosis-inducing drugs such as erastin, sorafenib, and buthionine sulfoximine (BSO), enhance NRF2 activity which contributes to ferroptosis resistance partly through increased expression of genes involved in heme, iron, and ROS metabolism [58]. The use of an isogenic lung cancer cell model also identified NRF2 as an important factor for erastin resistance [59]. NRF2 can upregulate SLC7A11 expression [60,61] and SLC7A11 overexpression can partially rescue the effects of NRF2 knockdown [62]. NRF activity was also essential for preventing ferroptosis of inner, matrix-detached cells in 3D spheroids models [63]. A recent study showed that alternative reading frame (ARF), a well-established tumor suppressor, is critical for NRF activity [64]. Mechanistically, ARF can bind directly to NRF2 and inhibit NRF2-mediated transcription activation of target genes. Consequently, ARF expression sensitizes cells to ferroptosis and this is independent of the function of ARF in activating p53 pathway.

SLC7A11 can undergo epigenetic regulation by the deubiquitinating enzyme BAP1 [3]. Despite *BAP1* being a well-known tumor suppressor gene, the mechanisms by which BAP1 exerts its tumor suppressor function remain unclear. Through cancer genomic analyses, it was shown that SLC7A11 and BAP1 expression were inversely correlated in cancer. Further mechanistic studies demonstrated that BAP1 suppresses SLC7A11 expression partly by deubiquitinating histone 2A ubiquitination to promote ferroptosis. Importantly, cancer-associated BAP1 mutations are defective in SLC7A11 regulation and ferroptosis, suggesting that SLC7A11-mediated ferroptosis has a role in the tumor-suppressive function of BAP1.

Several studies have described post-translational regulation mechanisms of SLC7A11. First, the ubiquitin hydrolase, OTU domain-containing ubiquitin aldehyde-binding protein 1 (OTUB1), has been demonstrated to modulate the stability of SLC7A11 by direct interaction [65]. The enhanced stability of SLC7A11 by OTUB1 was supported by the cancer stem cell marker, cluster of differentiation 44 (CD44). This may be related to the finding that SLC7A11 and CD44 variant can form a complex with the oncogenic C-terminal subunit of mucin-1 (MUC1-C) [66]. It will be of interest to examine whether these interactions are relevant in cancer-specific contexts. Second, it was established that beclin 1 (BECN1) promotes ferroptosis by forming a complex with SLC7A11 that inhibits system X_c_^−^ activity [67]. It was demonstrated that AMP-activated protein kinase (AMPK)-mediated phosphorylation of BECN1 was required for this activity and activation of BECN1 synergized with erastin to induce ferroptosis both in vitro and in vivo.

### 3.2. Transsulfuration Pathway

Although utilizing cystine that enters the cells through system X_c_^−^ is the dominant source for maintaining cysteine levels for glutathione biosynthesis, it is not the only existing pathway. Another way to maintain cysteine levels is through the transsulfuration pathway, which utilizes methionine to produce cysteine that can be further used to synthesize glutathione. The transsulfuration pathway generates homocysteine from methionine, which can either be synthesized into methionine, to repeat the cycle, or cystathionine. From cystathionine, it can be synthesized into cysteine, as an alternate source to produce glutathione when system X_c_^−^ is blocked. This pathway can prevent cells from undergoing ferroptosis as shown in the following study. From an unbiased genome-wide screen to identify genes necessary for erastin-induced ferroptosis, *cysteinyl-tRNA synthetase* (*CARS*) was identified as an inducer of ferroptosis [68]. It was shown that CARS knockdown led to an accumulation of cystathionine, an intermediate of the transsulfuration pathway, resulting from the upregulation of genes involved in serine biosynthesis and transsulfuration. This explains why knockdown of CARS can suppress erastin-induced, but not RSL3- or BSO-induced ferroptosis. In addition, the role DJ-1, an oxidative stress sensor, in suppressing ferroptosis through the regulation of the transsulfuration pathway was observed [69]. Mechanistically, the presence of DJ-1 prevents the binding of S-adenosyl homocysteine hydrolase (SAHH), the only known enzyme that generates homocysteine from S-adenosyl-L-homocysteine (SAH), with its negative regulator adenosylhomocysteinase like 1 (AHCYL1). Therefore, in the context of DJ-1 depletion, cells are sensitized to ferroptosis and can synergize with ferroptosis inducers.

### 3.3. Glutathione

Glutathione plays an important role in cell survival by circulating between the reduced or oxidized form to function as an electron donor or acceptor. Such a role is critical as it takes part in processes such as protein folding and antioxidant defense. In the context of ferroptosis, glutathione has an important function to maintain GPX4 activity and, as such, regulation of glutathione homeostasis within cells can influence ferroptosis sensitivity. For example, ΔNp63α, the major isoform of p63, was shown to be a central regulator of redox homeostasis through the control of glutathione biogenesis, utilization, and regeneration by directly interacting with the glutathione biosynthesis enzymes GCL and GSS, along with the NADPH-synthesizing enzyme, isocitrate dehydrogenase 1 (IDH1) [70]. Overexpression of ΔNp63α protected cells from ferroptosis in a p53-independent mechanism. In addition, ΔNp63α promoted metastasis in certain contexts and it will be of interest to examine whether suppression of ferroptosis can contribute this phenotype. Not only is the biosynthesis of glutathione critical for regulating ferroptosis but the efflux of glutathione may also be important. In a genome-wide screen using a glutathione probe to identify genes that regulate intracellular glutathione levels, *ATP binding cassette family member 1* (*ABCC1*), which encodes multidrug resistance protein 1 (MRP1), was found to negatively regulate glutathione abundance by inducing efflux out of the cell [71]. This MRP1-mediated glutathione efflux can sensitize cells to ferroptosis but confer a multidrug resistance at the same time as it can export other chemotherapeutic drugs.

### 3.4. GPX4

As mentioned above, ferroptosis can be induced with the direct inhibition of GPX4 in some contexts [7]. As a member of the GPX family, GPX4 shares the common basic function of reducing peroxides by utilizing glutathione or other thiol-containing compounds [72]. However, GPX4 is unique in directly inhibiting phospholipid hydroperoxides in membranes and lipoproteins and is, therefore, the only GPX essential for development [73]. Further confirming the importance of GPX4, it was shown that drug-tolerant persister cells are vulnerable to GPX4 inhibition [11]. This was caused by the global downregulation of antioxidant genes and decreased glutathione and NADPH levels, leading to a heightened dependence on GPX4 activity. This indicates that GPX4 may be an ideal target for therapeutic methods to prevent tumor relapse.

Several mechanisms of GPX4 regulation in cancer cells have been observed including regulation by heat shock 70 kDa protein 5 (HSPA5), a molecular chaperone found primarily in the endoplasmic reticulum [74]. The upregulation of HSPA5, caused by activating transcription factor 4 (ATF4), inhibits GPX4 degradation through direct interaction. Inhibition of this pathway can sensitize cells to ferroptosis and enhance gemcitabine sensitivity. In a recent study, it was found that mutation of IDH1 sensitizes cells to ferroptosis [75]. IDH1/2 mutations are observed in several cancer types and lead to the overproduction of the oncometabolite 2-hydroxyglutarate (2-HG), which can competitively inhibit various α-ketoglutarate (α-KG)-dependent dioxygenases causing epigenetic regulation or genetic instability. Both the ectopic expression of IDH1 mutation or the treatment of cell-permeable 2-HG led to an increase in lipid ROS and ferroptosis accompanied by reduced GPX4 levels and depletion of glutathione. Whether these effects are directly regulated by mutant IDH1 or 2-HG or a result of indirect mechanisms will be of interest for future studies. An additional mechanism of GPX4 regulation is by a compound named FIN56 which was identified as a specific inducer of ferroptosis that partially acts by inducing GPX4 degradation [76].

### 3.5. Selenium

As GPX4 is a selenocysteine-containing enzyme, selenium and its incorporation into the amino acid selenocysteine through the selenocysteine biosynthesis pathway are important for ferroptosis [77]. Selenocysteine is similar to cysteine but with selenium in the place of the usual sulfur and is essential for the function of selenoproteins such as GPX4 and thioredoxin reductases [78]. Due to only having small dissimilarities, most selenoproteins can also exist as homologs that hold cysteine. In a detailed study comparing selenocysteine- and cysteine-containing GPX4, it was shown that the selenolate-based catalysis was dispensable for normal embryogenesis [78]. However, the selenocysteine-containing GPX4 was an essential factor for the prevention of lethal epileptic seizure caused by certain types of interneurons, suggesting an indispensable role for selenium. Mechanistically, the selenocysteine utilization by GPX4 confers resistance to an overload of lipid peroxidation and subsequent ferroptosis. This protective role of selenium was further implicated in another study that demonstrated that the supplementation of selenium prevents neurons from undergoing ferroptosis by upregulating a transcriptional adaptive program [79]. Specifically, selenium activates the transcription factors transcription factor AP-2 gamma (TFAP2c) and specificity protein 1 (Sp1) to upregulate GPX4 along with other genes of this transcriptional program, called the selenome. A single dose of selenium was able to induce this adaptive mechanism to drive GPX4 expression, protect neurons, and improve behavior in a hemorrhagic stroke model. However, selenium metabolism has a double-edged effect, in that selenide, an intermediate produced during selenocysteine biosynthesis, is toxic to cells. This causes some cancer cells to become dependent on selenophosphate synthetase 2 (SEPHS2) to detoxify selenide [80]. Similar to the observation that proliferation and survival of cells did not require selenoproteins as long as there was residual cysteine-containing GPX4 function [78], SEPHS2 was not essential for the survival of normal cells under basal conditions. Especially, the elevated expression of SLC7A11 in selenophilic cancer cells allows for the increased import of cystine, which is reduced to cysteine and then exported. This leads to the extracellular accumulation of thiols from cysteine and reduction of selenite to volatile selenide, inducing import into the cell and toxicity. Such findings suggest that SLC7A11 generates a dependency on SEPHS2 and targetable vulnerability and further highlight the role of selenium metabolism in cancer.

## 4. Lipid Metabolism

**Figure 3:**
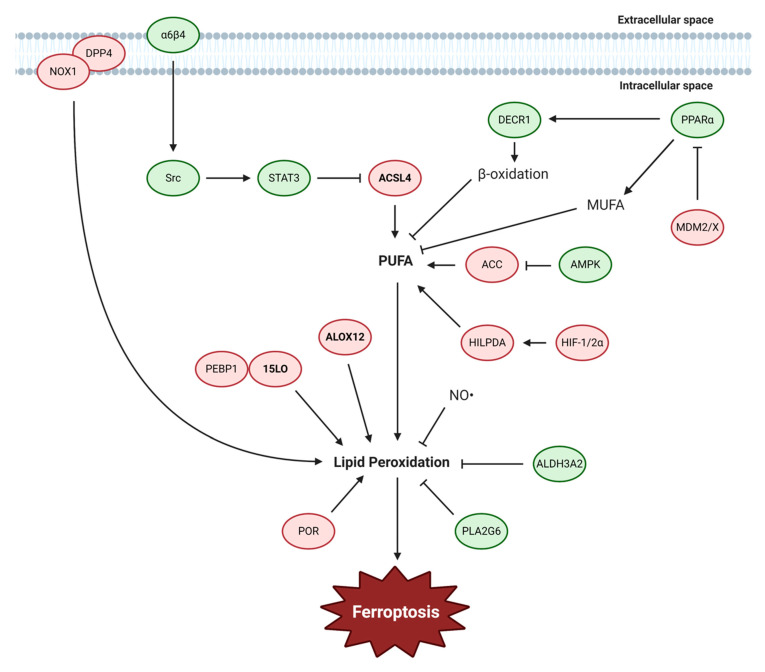
Lipid metabolism regulates ferroptosis. The major stages of lipid metabolism that regulate ferroptosis are the generation of PUFA and the induction of lipid peroxidation. ACSL4 is the major enzyme that generates PUFAs which drive ferroptosis. In the case of lipid peroxidation, ALOX and other factors can contribute to the production of lipid peroxides. Ferroptosis inhibiting and inducing factors are indicated in green and red, respectively. (Abbreviations: 15LO, 15-lipoxygenase; ACC, acetyl-CoA carboxylase; ACSL4, acyl-CoA synthetase long-chain family member 4; ALDH3A2, aldehyde dehydrogenase 3 family member A2; ALOX, arachidonate 5-lipoxygenase; AMPK, 5′ AMP-activated protein kinase; DECR1, 2,4-dienoyl-CoA reductase 1; DPP4, dipeptidyl-peptidase 4; HIF-1/2α, hypoxia-inducible factor 1/2α; HILPDA, hypoxia inducible lipid droplet associated; MDM2/X, murine double minute family members 2/X; MUFA, monounsaturated fatty acids; NO, nitrogen oxide; NOX1, NADPH oxidase 1; PEBP1, phosphatidylethanolamine binding protein 1; PLA2G6, phospholipase A2 group VI; POR, P450 oxidoreductase; PPARα, peroxisome proliferator-activated receptor alpha; PUFA, polyunsaturated fatty acids; STAT3, signal transducer and activator of transcription 3).

The execution of ferroptosis is ultimately determined by production of electrophilic species, enabling the accumulation of lipid peroxides. Although other pathways including the iron metabolism and cyst(e)ine-glutathione-GPX4 axis contribute to ferroptosis, eventually these pathways converge to accumulation of lipid peroxidation suggesting that lipid peroxides act as the final executor of ferroptosis. There are two major steps to how lipid metabolism mainly contributes to ferroptosis. The first step is the generation of PUFAs and the second step is the oxidation of these lipids. For these processes, the class of lipids is equally important as the production of electrophilic species. Lipids can be classified into many categories, such as fatty acids, glycerolipids, or glycerophospholipids. Fatty acids, which are the main type of lipids associated with the lipid peroxide species generated during ferroptosis, are composed of hydrocarbon chains ending in carboxyl groups and can be categorized into two groups, saturated fatty acids and unsaturated fatty acids. Among unsaturated fatty acids, PUFAs are fatty acids that have more than one carbon=carbon double bond which make them susceptible to attack by free radicals for the induction of lipid peroxidation. Common examples of PUFAs are arachidonic acid (AA) (C20:4) and linoleic acid (C18:2) which have four and two double bonds, respectively. These long PUFAs can be produced by members of the ACSL family, especially ACSL4, which prefers AA as a substrate [81]. ACSL4 acts as the main contributor of ferroptosis whereas the other family members are not closely associated with ferroptosis [82,83]. The abundance of PUFAs is a determining factor for the degree of lipid peroxidation, and thus ferroptosis, that can occur in cells. Lipid peroxidation can be caused by free radicals formed from Fenton reactions, radicals produced from nitric oxide synthase, or directly oxidized by lipoxygenases [84]. Therefore, the regulation of either the lipid biosynthesis process or oxidation mechanisms can lead to the regulation of ferroptosis. Here, we discuss the studies that have shown mechanisms of lipid metabolism regulating ferroptosis (Figure 3).

### 4.1. PUFA Biosynthesis and ACSL4

Production of PUFA can serve as the first step for induction of ferroptosis in a lipid metabolic pathway. It has been suggested that specific types of phospholipids, such as phosphatidylethanolamines (PEs), are preferentially oxidized in ferroptosis [85]. It was shown in a recent study that AMPK activation inhibits ferroptosis though the regulation of PUFA generation [86]. AMPK activation contributes to ferroptosis inhibition by inhibiting acetyl-CoA carboxylase (ACC), which produces malonyl-CoA that is important for fatty acid synthesis, and abrogates PUFA synthesis. These results show that modulating the level of PUFA can be an effective mechanism to regulate ferroptosis. In a previous study, AMPK has also been shown to promote ferroptosis by blocking system X_c_^−^ activity [67], indicating that AMPK-mediated ferroptosis regulation is context-dependent. Interestingly, treatment of exogenous monounsaturated fatty acids (MUFAs), such as oleic acid, are able to potently inhibit ferroptosis by displacing PUFAs from the plasma membrane [84,87]. This pathway can be regulated by the PI3K-AKT-mTOR pathway, which can suppress ferroptosis by production of MUFAs via SREBP-mediated lipogenesis [88]. MDM2, an E3 ligase, is a well-known inhibitor of p53 [89] and MDMX can function to enhance MDM2 activity through complex formation. However, in a p53-independent manner, the MDM2-MDMX complex regulates lipid metabolism in cells to increase both MUFAs, which inhibit ferroptosis, and coenzyme Q_10_ (CoQ_10_), a key antioxidant component, most likely through the activity of PPARα, a master regulator of lipid metabolism [90].

Several cancer types have been demonstrated to exhibit modulation in lipid metabolism relevant for ferroptosis. For example, in renal clear cell carcinomas, which are characterized by aberrant lipid and glycogen accumulation, it was found that hypoxia-inducible factor 2 alpha (HIF2-α) can drive the production of PUFAs, through the activity of hypoxia-inducible, lipid droplet-associated protein (HILPDA) [91]. This makes these tumors intrinsically more vulnerable to ferroptosis-inducing agents, revealing therapeutic insights to these aggressive malignancies. Importantly, a therapy resistant high-mesenchymal cell state that is characterized by enhanced activity of PUFA synthesis enzymes can render these cancer cells sensitive to ferroptosis [10]. These mesenchymal cells express zinc finger E-box-binding homeobox 1 (ZEB1) which can activate PPARγ, the master regulator of lipid metabolism, causing cells to become vulnerable to GPX4 inhibition. Similarly, in the case of gastric cancer, the expression of elongation of very long-chain fatty acid protein 5 (ELOVL5) and fatty acid desaturase 1 (FADS1), which are enzymes of the PUFA biosynthesis pathway, determine ferroptosis sensitivity [92]. These genes were upregulated in mesenchymal-type gastric cancer cells leading to their sensitization to ferroptosis. In contrast, prostate cancers exhibit enhanced fatty acid oxidation (FAO) and increased expression of 2,4 dienoyl-CoA reductase 1 (DECR1), a rate-limiting enzyme in β-oxidation of PUFA, in malignant prostate tissues compared to nonmalignant tissues [93]. Targeting of DECR1 not only resulted in a disruption of PUFA β-oxidation but accumulation of PUFAs in phospholipids that caused cells to become susceptible to ferroptosis.

ACSL4 is one of the major PUFA-modifying enzymes, responsible for the esterification of CoA to free fatty acids for oxidation or lipid biosynthesis. ACSL4 preferentially utilizes long-chain PUFAs such as AA and adrenic acid (AdA) to generate arachidonoyl-CoA (AA-CoA) and adrenoyl-CoA (Ada-CoA), respectively, which are used as substrates for lipid peroxidation in ferroptosis. This activity of ACSL4 has been identified by multiple groups as a targetable mechanism in preventing ferroptosis [82,83,94]. Lysophosphatidylcholine acyltransferase 3 (LPCAT3), which catalyzes the insertion of acylated arachidonic acid into membrane phospholipids, was also identified among the genes involved in ferroptosis [82], but it is unclear whether LPCAT3 plays a widespread role in regulating ferroptosis in various cell types. Physical interactions may be an important regulator for ACSL4 as extracellular matrix (ECM) detachment can act as an inducer of ferroptosis [95]. Mechanistically, the α6β4 integrin activates Src which represses ACSL4 expression through the activity of STAT3. It will be of interest to understand whether this mechanism is associated with NRF2-mediated survival in matrix detachment [63] and how different types of cell death, such as anoikis, crosstalk in ECM detachment contexts.

### 4.2. ALOX

The function of lipoxygenases, iron-containing enzymes that catalyze the oxygenation of PUFAs, to produce hydroperoxides can drive the ferroptosis process [84]. In humans there are six types of lipoxygenases: ALOX5, ALOX12, ALOX12B, ALOX15, ALOX15B, and ALOXE3, and several types have been implicated in ferroptosis. In the case of ALOX15, this lipoxygenase normally uses free PUFAs as substrates to make 12-hydroperoxyeicosatetraenoic acid (HpETE) and 15-HpETE. Importantly, the binding of ALOX15 with phosphatidylethanolamine-binding protein 1 (PEBP1) can change its substrate specificity from free PUFA to PUFA-PE [96]. This allows for the generation of HpETE-PEs that can act as ferroptosis signals. In a following study, it was shown that ferrostatin-1, a ferroptosis-specific inhibitor, binds to the PEBP1/ALOX15 complex and disrupts the catalytically required allosteric motions [97]. Induction of spermidine/spermine N^1^-acetyltransferase 1 (SAT1), a rate-limiting enzyme in polyamine metabolism and transcriptional target of p53, leads to increased ALOX15 expression and ALOX15 inhibition can completely rescue SAT1-induced ferroptosis. [98]. The phospholipase A2 group VI (PLA2G6) hydrolyzes the hydroperoxy-phosphatidylethanolamine (Hp-PE) species that are implicated in ferroptosis into lyso-PE and oxidized fatty acid, thereby blocking the accumulation of lipid peroxides to initiate ferroptosis [99]. This activity was not apparent in the presence of intact GPX4 activity, suggesting that it may function as a secondary defense mechanism. In the case of p53-mediated ferroptosis induced through ROS generation by tert-Butyl hydroperoxide (TBH) treatment, it was shown that ALOX12 activation was necessary [100]. Here, ALOX12 was found to bind to SLC7A11 and inhibited its lipoxygenase activity. However, ALOX12 was found to be dispensable for erastin- or RSL3-induced ferroptosis, suggesting that various ferroptosis-inducing pathways may utilize distinct lipoxygenases. Lipid peroxidation pathways leading to ferroptosis may also regulate cell death in various cell types. M1-polarized macrophages and microglia express iNOS-derived NO•, which possess antiferroptotic effect most likely by inhibiting ALOX activity [101]. As NO• can diffuse through membranes, it was shown that this ferroptosis-protective activity of macrophages could be passed on to cocultured epithelial cells. It was also shown that the NO•-mediated ferroptosis inhibition can compensate for GPX4 depletion in certain contexts.

Other mechanisms of lipid peroxidation may exist as shown in colorectal cancer cells, in which p53 acts as a negative regulator of ferroptosis by blocking dipeptidyl-peptidase-4 (DPP4) activity through a transcription-independent mechanism [102]. In these cells, p53 deletion leads to reduced SLC7A11 mRNA, suggesting that the role of p53 in regulating ferroptosis is context-dependent. Instead, p53 in colorectal cancer specifically communicates with DPP4 that interacts with NADPH oxidase 1 (NOX1), which was shown to contribute to the source of ROS in erastin-treated cells [26]. In addition, aldehyde dehydrogenase 3 family member A2 (ALDH3A2), an enzyme that oxidizes long-chain aldehydes to prevent oxidative damage, is critical to prevent ferroptosis in leukemia cells but not normal hematopoietic cells [103]. ALDH3A2 depletion leads to altered lipid composition and inability to downregulate lipid ROS which causes ALDH3A2 depletion to be synergistic with GPX4 inhibition. Lastly, in a genome-wide CRISPR-Cas9 suppressor screen to identify ferroptosis regulators, *cytochrome P450 oxidoreductase* (*POR*) was found to be a major contributor to ferroptosis [104]. Although the exact mechanism of how POR functions remains to be established, it was shown that POR regulates ferroptosis by promoting lipid peroxidation presumably with an enzymatic partner that acts as an electron acceptor.

## 5. Other Metabolic Pathways

### 5.1. FSP1-Coenzyme Q_10_ Pathway

**Figure 4:**
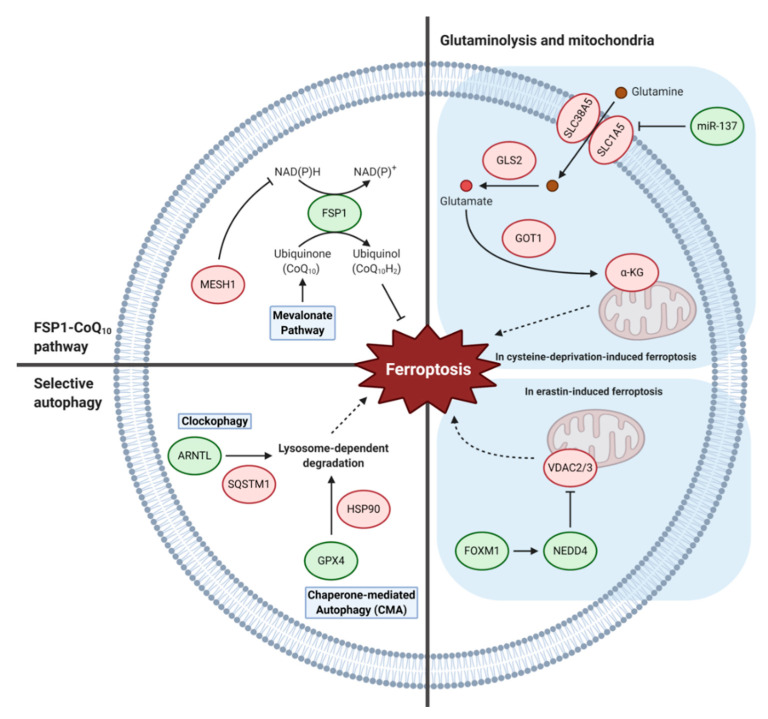
Other metabolic pathways that regulate ferroptosis. Aside from the main metabolic pathways, there are additional pathways that can regulate ferroptosis such as the FSP1-CoQ_10_ pathway, glutaminolysis and mitochondria-related pathway, and selective autophagy pathways. Ferroptosis inhibiting and inducing factors are indicated in green and red, respectively. (Abbreviations: α-KG, α-ketoglutarate; ARNTL, aryl hydrocarbon receptor nuclear translocator like; FOXM1, forkhead box protein M1; FSP1, ferroptosis suppressor protein 1; GLS2, glutaminase 2; GOT1, glutamate oxaloacetate transaminase 1; GPX4, glutathione peroxidase 4; HSP90, heat shock protein 90; MESH1, metazoan SpoT homologue 1; NADH, nicotinamide adenine dinucleotide; NADK, NAD kinase; NADPH, nicotinamide adenine dinucleotide phosphate NEDD4, neuronal precursor cell-expressed developmentally downregulated 4; SLC1A5, solute carrier family 1 member 5; SLC38A5, solute carrier family 38 member 5; SQSTM1, sequestosome 1; VDAC2/3, voltage-dependent anion channels 2 and 3).

Two recent studies have reported a critical ferroptosis-inhibiting role of ferroptosis suppressor protein 1 (FSP1), previously known as apoptosis-inducing factor mitochondrial 2 (AIFM2), revealing an additional pathway in parallel to the GPX4 pathway that cells utilize to resist from undergoing ferroptosis [105,106]. In one study, a CRISPR-Cas9 screen showed that FSP1 functions in a synthetic lethal manner with GPX4 [105] and in another study, FSP1 expression was found to complement the loss of GPX4 [106]. It was further shown that the myristoylation of FSP1 mediates the recruitment of this protein to the plasma membrane, where it performs its function as an oxidoreductase that reduces CoQ_10_, also called ubiquinone, by NAD(P)H. The reduced form, CoQ_10_H_2_, or ubiquinol, acts as a lipophilic radical-trapping antioxidant that halts the propagation of lipid peroxides. In addition, FSP1 expression levels correlated with ferroptosis resistance in many cancer cell lines suggesting that FSP1 expression levels may predict the efficacy of ferroptosis-inducing drugs in cancers. It will be important for future studies to understand how the cyst(e)ine-glutathione-GPX4 and FSP1-CoQ_10_ pathway work together to prevent cells from undergoing ferroptosis and whether the concomitant targeting of these two pathways will be an effective method for cancer therapy. In addition, treatment of statins, which are inhibitors of the enzyme 3-hydroxy-3-methylglutaryl-coenzyme A (HMG-CoA) reductase, can be used to block the mevalonate pathway that functions to support the production of CoQ_10_. Indeed, a ferroptosis-inducing agent, FIN56, depletes CoQ_10_ by modulating the activity of squalene synthase, an enzyme in the mevalonate pathway, and sensitizes cells to ferroptosis [76]. The mevalonate pathway can also affect the activity selenocysteine tRNA, essential for GPX4 synthesis, through isopentenyl pyrophosphate, an intermediate of the mevalonate pathway. In a recent study, NADPH levels and oxidoreductases that use NAD(P)^+^ and NAD(P)H were identified as biomarkers of ferroptosis sensitivity [107]. Additionally, in another study, metazoan SpoT homologue 1 (MESH1) was identified as a NADPH phosphatase that induces ferroptosis through the depletion of NADPH [108]. The survival advantage of MESH1 depletion can be reversed by simultaneous depletion of NAD kinase, suggesting that preserving cellular NADPH levels is critical to prevent cells from undergoing ferroptosis. As NADPH is used by FSP1, for reduction of CoQ_10_, and glutathione reductase, for reduction of glutathione, NADPH depletion may have multiple regulation inputs into the ferroptosis pathway (Figure 4).

### 5.2. Glutaminolysis and Mitochondria

Two serum factors, TF and glutamine, have recently been shown to act as inducers of ferroptosis in the context of cystine deprivation [109]. Inhibition of the glutamine transporter, glutaminase 2 (GLS2), or the glutamate oxaloacetate transaminase 1 (GOT1) led commonly to the abrogation of ferroptosis suggesting that glutaminolysis plays an important role in ferroptosis. In support of these results, it was shown that miR-137 negatively regulates ferroptosis by directly targeting the glutamine transporter SLC1A5 in melanoma cells [110]. Ectopic expression of miR-137 suppressed SLC1A5, resulting in decreased glutamine uptake, whereas knockdown of miR-137 increased the anticancer activity of erastin by enhancing ferroptosis. Additionally, a single-nucleotide polymorphism in *TP53*, a S47 variant, was shown to be impaired for tumor suppression as S47 heterozygous and homozygous mice are predisposed to hepatocellular carcinoma and other cancers [111]. This S47 variant manifests normal functions for most known p53 functions but has an impaired ability to transactivate GLS2 and undergo ferroptosis which may partially explain the tumor-prone phenotype of the S47 mice. These studies suggest that regulation of glutaminolysis can modulate ferroptosis sensitivity.

A major function of glutaminolysis is to maintain the TCA cycle through anaplerosis, by generating the metabolite α-KG. It was recently shown that α-KG can replace the function of glutamine for cystine deprivation-induced ferroptosis suggesting that the mitochondrial TCA cycle participates in this process [4]. Similarly, other metabolites of the TCA cycle, succinate, fumarate, and malate, can also replace glutamine for ferroptosis in these contexts. It was further shown that glutaminolysis and the TCA cycle drive mitochondrial membrane potential hyperpolarization which is associated with increased lipid peroxidation and ferroptosis. However, glutamine and mitochondria are dispensable for GPX4 inhibition-induced ferroptosis suggesting there are derivations of pathways leading to ferroptosis. Another role for the mitochondria in ferroptosis is through the voltage-dependent anion channels 2 and 3 (VDAC2/3) which have been previously identified as direct targets of erastin [112] and shown to be necessary, but not sufficient, for erastin-induced ferroptosis [26]. Recently, the E3 ligase neuronal precursor cell-expressed developmentally downregulated 4 (NEDD4) was shown to induce the degradation of VDAC2/3 during erastin- but not RSL3-induced ferroptosis [113]. The transcription factor forkhead box protein M1 (FOXM1) is responsible for the increase in NEDD4 expression in response to erastin and can lead to inhibition of ferroptosis through subsequent VDAC2/3 degradation.

### 5.3. Selective Autophagy

As mentioned above, selective autophagy, in the form of ferritinophagy, can regulate ferroptosis by the degradation of ferritin and subsequent increase of intracellular iron levels [38]. Aside from ferritinophagy, several other types of selective autophagy have also been found to regulate ferroptosis. Firstly, clockophagy is the autophagy-dependent degradation of aryl hydrocarbon receptor nuclear translocator like (ARNTL), a central component of the mammalian circadian clock [114]. It was shown that ARNTL was selectively degraded, through the activity of SQSTM1 as a cargo receptor, with treatment of GPX4 inhibitors but not SLC7A11 inhibitors. Mechanistically, ARNTL degradation promotes ferroptosis by inhibiting HIF1α-dependent fatty acid uptake and lipid storage. Secondly, chaperone-mediated autophagy (CMA) is another type of selective autophagy that utilizes chaperones for the degradation of specific proteins based on their sequences. In a recent study, the compound 2-amino-5-chloro-N,3-dimethylbenzamide (CDDO) was shown to inhibit ferroptosis by blocking CMA-mediated degradation of GPX4 [115]. Mechanistically, erastin treatment leads to the inhibition of heat shock protein 90 (HSP90), which can increase lysosomal-associated membrane protein 2A (LAMP2A)- and HSP70-dependent CMA. These studies suggest that multiple selective autophagy pathways may interact to determine ferroptosis susceptibility in cancer cells.

## 6. Conclusions and Perspectives

Ferroptosis features accumulation of lipid peroxides and the dependency on multiple metabolic pathways. Recent advances have provided insights into the precise molecular mechanisms of ferroptosis, particularly its relationship with cellular metabolism. The relevance of ferroptosis in various human diseases have been established, supporting the need for future studies to investigate the role of ferroptosis in these diseases as well as the development of better methods to modulate ferroptosis in vivo. Interestingly, it has been shown that iron accumulation increases throughout the lifespan of *Caenorhabditis elegans* and blocking ferroptosis can lead to an increase in lifespan suggesting that limiting ferroptosis may also promote healthy aging [116]. Furthermore, intercellular interactions may be a critical factor in regulating ferroptosis as a characteristic of ferroptosis is that this death process propagates to neighboring cells in a wave-like manner [29,117]. This pattern has been observed in various systems [118,119] and may provide insights to how ferroptosis can be controlled in a cell population. However, many critical questions remain in the study of ferroptosis. How do the central metabolic processes in ferroptosis, namely that of iron, cyst(e)ine, glutathione, or lipids, communicate with each other to dictate the output of survival versus ferroptosis? What are the metabolic markers that can be used to dictate ferroptosis sensitivity in cancer cells? Answers to these questions will help elucidate the ferroptosis pathway and will be instrumental in translating this knowledge of basic cell biology to clinical settings.

## Data Availability

Not applicable.
